# Ten simple rules for good model-sharing practices

**DOI:** 10.1371/journal.pcbi.1012702

**Published:** 2025-01-10

**Authors:** Ismael Kherroubi Garcia, Christopher Erdmann, Sandra Gesing, Michael Barton, Lauren Cadwallader, Geerten Hengeveld, Christine R. Kirkpatrick, Kathryn Knight, Carsten Lemmen, Rebecca Ringuette, Qing Zhan, Melissa Harrison, Feilim Mac Gabhann, Natalie Meyers, Cailean Osborne, Charlotte Till, Paul Brenner, Matt Buys, Min Chen, Allen Lee, Jason Papin, Yuhan Rao

**Affiliations:** 1 Kairoi Ltd., London, United Kingdom; 2 SciLifeLab, Stockholm, Sweden; 3 University of California San Diego, La Jolla, California, United States of America; 4 San Diego Supercomputer Center, La Jolla, California, United States of America; 5 US-RSE, Oakland, California, United States of America; 6 Arizona State University, Tempe, Arizona, United States of America; 7 Public Library of Science, San Francisco, California, United States of America; 8 Netherlands Institute of Ecology, Wageningen, the Netherlands; 9 Oak Ridge National Laboratory, Oak Ridge, Tennessee, United States of America; 10 Helmholtz-Zentrum Hereon, Geesthacht, Germany; 11 Heliophysics Digital Resource Library, Greenbelt, Maryland, United States of America; 12 NASA Goddard, Greenbelt, Maryland, United States of America; 13 Royal Netherlands Institute for Sea Research, Texel, the Netherlands; 14 European Molecular Biology Laboratory, Hinxton, United Kingdom; 15 Johns Hopkins University, Baltimore, Maryland, United States of America; 16 University of Notre Dame, Notre Dame, Indiana, United States of America; 17 University of Oxford, Oxford, United Kingdom; 18 Linux Foundation, San Francisco, California, United States of America; 19 Datacite, Hanover, Germany; 20 Nanjing Normal University, Nanjing, China; 21 University of Virginia, Charlottesville, Virgina, United States of America; 22 North Carolina State University, Raleigh, North Carolina, United States of America; Carnegie Mellon University, UNITED STATES OF AMERICA

## Abstract

Computational models are complex scientific constructs that have become essential for us to better understand the world. Many models are valuable for peers within and beyond disciplinary boundaries. However, there are no widely agreed-upon standards for sharing models. This paper suggests 10 simple rules for you to both (i) ensure you share models in a way that is at least “good enough,” and (ii) enable others to lead the change towards better model-sharing practices.

## Introduction

Computational advancements enable scientific communities to better understand and communicate complex natural and social phenomena. Scientific practices have also evolved in light of the need for more dialogue among and between disciplines to study the intricate web of relationships between diverse objects of scientific inquiry. At the intersection of these technologies and scientific practices sits the open science movement, making research processes and outputs available to a wider audience. The present article suggests 10 rules for sharing computational models according to open science standards.

Let us begin with a working definition of “computational model,” which we use interchangeably with “model”:

C*onceptual constructs that are based on scientific theory and/or data, and embedded in a software setting to perform manipulations on input data and produce output data for the purpose of scientific advancement or policy development.*

The above definition of “model” is by no means perfect, but it begins to elicit some of the benefits of good model-sharing practices. From a scientific perspective, providing information about a model and its assumptions enables its reuse and scrutiny. Where a researcher discovers a model that is relevant to their work, the model’s openness allows for it to be adapted to the researcher’s work without needing to reverse-engineer the model, nor guess at its underlying assumptions. Sharing models also gives researchers the chance to promote their work according to common publication practices. Models that are shared following good practices can be understood by more academic audiences and cited in academic publications, allowing their creators and contributors to garner credibility among their peers. As well as peers, policy-makers and broader communities also tend to place greater trust in the outputs of models that are publicly accessible [1]. Indeed, evidence shows that open science practices increase the impact of data-driven research [2].

Sharing computational models does come with its own set of challenges. Firstly, there is a tension between more frequent requirements for model-sharing by funding agencies, and the minimal instructions on how to do so [3]. Secondly, the relevant cyberinfrastructure and standards for sharing models may be lacking, difficult to discover, or fragmented and hard to navigate. Thirdly, modeling often is an inherently multidisciplinary endeavor. This may complicate model-sharing because diverse disciplinary experts may need to come together to agree on a shared worldview for the project’s purpose [4]. What’s more, models often act as boundary objects between different scientific domains and diverse stakeholder groups. This creates several audiences with whom to share models, and each will have different interests. With this, we will use the following working definitions for 4 stakeholders of computational models:

- *Domain experts* are those with training in specific disciplines that use modeling to advance scientific understanding within their domains (e.g., biologists, economists, physicists, and anthropologists);
- *Model developers* are those with training in computer science, software engineering, and related fields that grant them the skills to develop the computational models that benefit diverse research domains;
- *Policy-makers* are those who develop and sometimes implement rules, regulations and plans from a governmental body; and
- *Archivists* are those dedicated to archiving research artifacts for a community and supporting model developers.

Other stakeholders will be introduced later on, including research software engineers (RSEs) and publishers. For now, it is worth noting that people with both domain expertise and model development skills are becoming increasingly common, such as in bioinformatics, oceanography, and heliophysics.

A fourth challenge to model-sharing relates with the need for careful planning, time, and effort. For example, the task of creating documentation that addresses the needs of all relevant stakeholders without a well-formulated standard can impose a large additional burden on researchers, especially for those on precarious contracts.

Finally, our definition of computational models incorporates various elements: conceptual constructs, data, metadata, and software. Meanwhile, the widely adopted open science principles of findability, accessibility, interoperability and reusability (FAIR) tend to focus on one or some of those elements. Indeed, the FAIR principles were originally applied to data [5] and have since been adapted for research software (FAIR4RS) [6] and machine learning (FAIR4ML) [7]. With this, the FAIR principles and their adaptations are applicable to some extent yet insufficient for models. Although we do not need to reinvent the wheel when developing or enacting good model-sharing practices thanks to the open science movement, work remains to be done.

**Fig 1:**
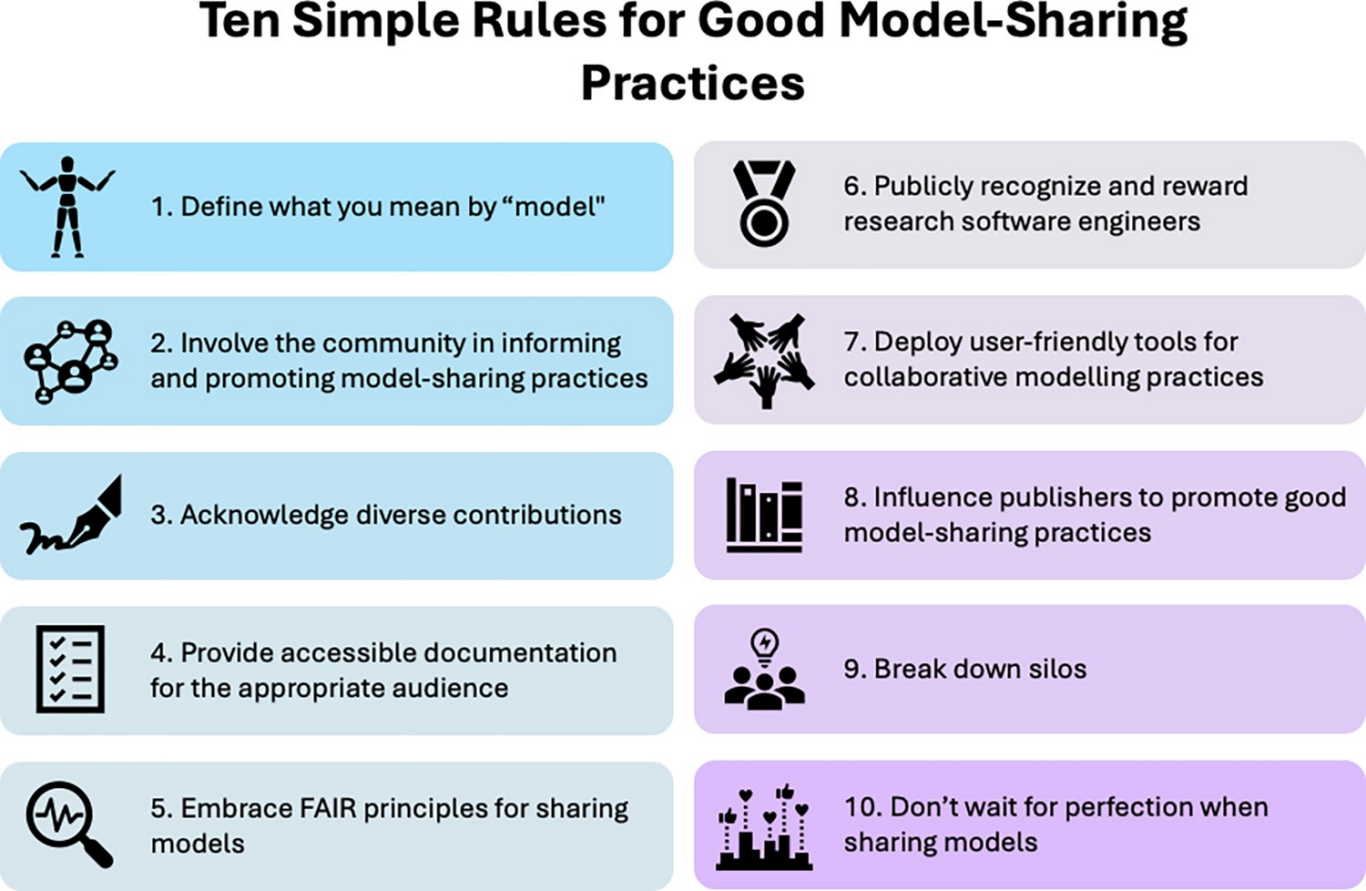
Ten simple rules for good model-sharing practices. The recommendations should be useful for widely different model developers and model stakeholders, and across public and private organizations. Three axes are worth keeping in mind when reading the recommendations. Firstly, both model developers in the ML space and those who do not work in ML should find value in the recommendations. Secondly, models developed for long-term impact and maintenance may gain from all the rules, while single-use models—models solely developed for analysis in one specific project—may only benefit from rules on contributor acknowledgement, metadata, and publication. Thirdly, the rules are split according to whether they describe changes you can work towards as an individual modeler (rules 1–6 and 10), or structural changes we believe you can play a part in (rules 7–9).

This is the backdrop of our 10 simple rules for good model-sharing practices. These recommendations result from a series of online workshops that took place between February and May 2024 [8]. The workshops were advertised to all members of the Open Modeling Foundation (OMF) and several networks that OMF executive committee members are a part of, including GO FAIR, US RSE, Open Life Science (OLS), All Tech Is Human, OpenSciency, and NASA. The workshops were agnostic to domains and modeling methods, touching on everything from physical, biological, and social systems, to equation-, agent-, and ML-based models. Each workshop focused on a topic that was brought to life by one or several experts, who then engaged in lively discussion with audience members (Fig 1).

The following 10 simple rules are designed to enable and promote good model-sharing practices that are tenable and flexible—this is why they are “good” and not “best” practices. We also note that we do not use “good” in its moral sense and that ethical considerations involved when sharing models and their diverse elements are beyond this paper’s scope. Incorporating some or all of the below practices into your model-sharing can significantly increase your work’s impact on the community, often resulting in increased citations, collaborations, opportunities, and funding.

### Rule 1: Define what you mean by “model”

Scientists and organizations can only understand one another and collaborate effectively when they use terms in similar ways [9–11]. A collision of terminology is highly likely, since models can be very diverse in nature. Therefore, when sharing your models—or even just speaking about models—clearly articulate what “model” means to yourself, your team, and your community.

Let’s take a moment to unpack this paper’s working definition of computational models:

*Conceptual constructs that are based on scientific theory and/or data, and embedded in a software setting to perform manipulations on input data and produce output data for the purpose of scientific advancement or policy development.*

The definition is discipline-agnostic to be inclusive of a great deal of modeling work that takes place in scientific research. The definition is also vague regarding models’ “software setting,” which could be for representing systems and their processes throughout time, eliciting correlations among large data sets, or something else. Finally, “scientific advancement or policy development” is what happens once there is a clear relationship between a model’s inputs and outputs, and insights can be gained and acted on. A model enables this, but its purpose must be more specific.

To have more productive discussions about the models we share, it is helpful to clarify their domain, type, and purpose.

- *Domain*: For which discipline was the model created? (E.g.: genomics, economics, or physics). Are there domain concepts used by the model that should be clarified or otherwise documented or domain-specific standards [12] being followed?
- *Type*: Describing a model’s type may elicit important features, such as the treatment of time and explainability of the outputs. One taxonomy of models defines 13 types of models: non-deterministic, deterministic, static, dynamic, discrete, continuous, stochastic, individual-based, population-based, logic, automata, black-box, and hybrid [13].
- *Purpose*: What is the purpose of the model being shared? Being explicit about this improves a model’s adoption and reuse statistics. Edmonds and colleagues suggest the 7 purposes: to anticipate, establish cause-effect chains, represent what is important, test hypotheses, communicate ideas, simulated processes, and further shared understanding [14].

The above lists of types and purposes are not exhaustive nor set in stone, and you may even feel your models fall into more than one of these domains, types, or purposes. It is worthwhile to carefully choose the best 1 or 2 items in each category that best align with your model. Clearly defining what you mean by “model” helps with understanding what essential components of the research are needed to describe, preserve, and cite your work. A clear definition supports transparency and replication of experiments, fostering a more collaborative and effective scientific environment.

### Rule 2: Involve the community in informing and promoting model-sharing practices

#### Community building is a key element to promoting good model-sharing practices. Individual model developers are embedded in larger communities and their behaviors are guided by community norms. You may shape those norms by involving your communities when sharing models

Establishing community engagement and buy-in is crucial for the open modeling efforts of any domain, especially early in the process. Where no defacto community modeling standards exist, we recommend:

1. Surveying your community for requirements, establishing the community’s needs and their unique challenges, as well as current good practices to avoid “reinventing the wheel”;
2. Engaging with the community to brainstorm potential or “strawman” solutions to those open modeling challenges;
3. Selecting a subset of community members who represent both respected experts in the field and those most willing and available to lead modeling standards efforts;
4. Being prepared to prioritize, as complete agreement is rarely attainable, and available resources are often insufficient to fund everyone’s efforts; and
5. Allowing emerging standards to be adopted by different communities and adapted to their contexts.

We find some of these elements present in the history of the Overview, Design concepts and Details (ODD) protocol. The protocol originally suggested a standardized format for describing agent-based models (ABMs) in ecology [15]. The protocol resulted from a workshop conducted in 2004 [16] and the contributions from 28 co-authors—this roughly covers recommendations 1-to-3 above. What’s more, as the protocol was more widely adopted, it was adapted to new applications, including more complex models [17] and other disciplines [18]—this is recommendation number 5.

Community involvement strategies don’t all look the same, but we can draw inspiration from relevant guidelines, established open science communities, and the role of early career researchers (ECRs). Regarding guidelines, consider the CARE Principles for Indigenous Data Governance [19]. For models, CARE means allowing for the community to benefit from their own and others’ contributions, giving the community a voice in controlling use and distribution of relevant models, and considering the ethics that relate to the use of the models (e.g., by documenting assumptions, known biases, and guidance for mitigating against identified risks).

Regarding open science communities, we may learn from the likes of FORRT, a community of over 600 people “raising awareness of the pedagogical implications of open and reproducible science in higher education” [20]. Other example organizations foster inclusive communities that value collaboration are 2i2c, The Carpentries, the Center for Scientific Collaboration and Community Engagement, Invest in Open Infrastructure, MetaDocencia, and OLS [21]. These initiatives demonstrate the effectiveness of community-focused strategies in promoting openness and reproducibility in research, offering valuable frameworks that can be adapted and implemented across various scientific disciplines.

For the long-term promotion of model-sharing practices, special attention should be given to ECRs. ECRs constitute the largest researcher community in most countries [22] and represent a new generation of researchers who have been trained during the software technology boom. This boom has laid the groundwork for open science FAIR model-sharing practices. However, there is increasing evidence that suggests the ECR community is not embracing open science nor sharing scientific artifacts enough [23,24]. This reluctance is understandable considering the “publish or perish” culture of academia. In addition to publishing in journals, sharing models according to good practices requires additional resources, which ECRs often lack.

Despite the challenges, many young researchers are commiting to open science through their own communities—such as DSOS (Data Science and Open Science) and AEMON-J (Aquatic Ecosystem MOdeling Network—Juniour) in the aquatic sciences—or spaces created by institutions—such as the Open Modeling Foundation’s Early Career Scholars Working Group [25,26]. Ultimately, ECRs are the torchbearers of transformative change, setting the norms of science in the near future. Therefore, it is crucial to provide sufficient support and guidance to this key community.

### Rule 3: Acknowledge diverse contributions

#### Computational modeling is usually a multidisciplinary endeavor. Contributions may be of very different types, involving model developers, domain experts, archivists, and policy-makers. With this, you must be ready to acknowledge such a great variety of contributors, and the Contributor Roles Taxonomy (CRediT) is one approach towards this goal

Often, modeling involves inputs from 2 types of parties: domain experts and model developers. While domain experts may provide the theory and data underpinning a model’s assumptions and an initial approach to formalizing a conceptual model, model developers would embed that domain expertise into software according to good practices. It would be unfair to publish a model in a way that did not attribute authorship to all those involved. However, generally, only those who write papers that are published in academic journals gain recognition through authorship. This matters because authorship is often the currency of the credit economy of science [27], and there are few journals that publish models [28].

One movement that has successfully challenged the status quo of academic publishing is the “Hidden REF” in the UK, which celebrates all research outputs, not disproportionately rewarding publications like the “research excellence framework” does [29]. This is a step in the right direction for model-sharing, as models are not generally peer-reviewed through academic publications. So, when sharing models, it is important to think carefully about whom to acknowledge and how.

Commonly, scientific contributions are recognized through authorship. Despite making scientific contributions, authorship is not typically given to RSEs or other people not contributing to publication texts. To resolve this issue and acknowledge such diverse contributions, publishers are progressively integrating the CRediT into their workflows and metadata systems [30]. Below, we outline 3 contributor roles that are particularly pertinent to the context of modeling:

- Data curation—which CRediT defines as activities related to training data annotation, cleaning, and maintenance—extremely time-consuming yet fundamental to model-production. Publicly available data sets for certain types of modeling (e.g., healthcare) are notably scarce. Furthermore, issues of missing or noisy data require preprocessing techniques that require special considerations for categorizing data (e.g., blood pressure data being “high,” “medium,” or “low” according to different clinical guidelines).
- Formal analysis necessitates computational or mathematical techniques for data analysis or synthesis. After data cleaning, analysis may include imputation (e.g., mean, median, or k-Nearest Neighbors) to fill in missing values based on extant data, data partitioning for training and testing, feature extraction and selection, hyperparameter optimization, dimensionality reduction, and performance metrics.
- Software development and related tasks are well covered by the “All Contributors” effort, which enables semi-automated contribution roles to be added for a person contributing to a GitHub repository [31]. However, modeling may require an additional contributor role for hardware, which can often be quite specific for the given model. Indeed, certain models can only run with sufficient computational power (e.g., a GPU as opposed to a CPU).

The structure required to recognize nontraditional contributions is growing. Initiatives such as CHAOSS are investigating methods to represent these contributions effectively for the benefit of research project health and individual recognition [32]. Repositories like Zenodo are incorporating contributor roles and increasingly adopting the CRediT taxonomy. It is now possible to easily add recognition for a person’s contribution to a GitHub repository using “All Contributors.” Additionally, specific communities are working to make such taxonomies more comprehensive, ensuring that all contributions receive appropriate credit (e.g., those in the geosciences) [33]. Even indicators of scientific credit are evolving. Consider DORA, a global initiative that aims to improve the ways in which scholar outputs are evaluated. DORA adopts a broad definition of *scientific output* that includes not only scholarly articles but also data sets, patents, and software [34].

### Rule 4: Provide accessible documentation for the appropriate audience

#### Make your model reusable and more impactful by providing documentation alongside it. By “documentation,” we mean a collection of documents and additional written material that describe a computational model across its entire life cycle and along with its underlying assumptions and scientific bases [35]. This helps communicate to diverse stakeholders why a model is worth using

Generally, models are more impactful where their users deem them to be (i) scientifically sound; (ii) relevant to the policy issue at hand; and (iii) the result of stakeholder engagement [36]. Accessible and thorough documentation is critical to model-sharing. In some cases, additional comments in documentation are needed to aid in understanding. Depending on the nature of the comments, they might be included in the code, in a public notes document where notes on the history of the model usage and stability are contributed, as structured metadata linked to the model’s metadata record, or as a combination of these. As a general rule, the more complex a model is, the more effort should be spent on creating its documentation, although metadata management technologies are becoming increasingly capable at streamlining such processes.

Documentation should contain valuable information for various audiences and be clearly organized. Efforts have already been made for making models more interpretable to different audiences. For example, “model cards” summarize, among other things, a model’s performance in different contexts and its intended scope [37]. They provide a helpful template for reaching audiences of different technical abilities. Meanwhile, the Model Openness Framework articulates the different components and their respective open licenses that are required for sharing deep learning models, how those objects interconnect, and the stakeholders that would be needed to support more robust model-sharing [38]. While such frameworks are comprehensive and require technical expertise to implement, they improve the model’s impact on downstream users.

At least 4 audiences benefit from tailored documentation: policy-makers, domain experts, archivists, and fellow model developers.

- Policy-makers gain from detailed documentation relating to potential policy impacts. Policy-informing models should be presented as such and accompanied by documentation that allow policy-makers to understand the science and articulate policy decisions based on the model [39]. Relevant documentation elicits assumptions, explains the contextual conditions and input parameters under which the model’s result should be considered valid, depicts the maturity of the model, and summarizes the underpinning science without using jargon.
- Domain experts benefit from documentation with more thorough explanations of the science underpinning a model, why it is a valid representation of its target, and why it is fit for purpose. Those explanations should include the jargon expected by experts in the science field but without any expectation of past modeling experience [40]. Indeed, research has shown that documentation targeted at domain experts may increase the trust they deposit in a model’s output [41].
- Archivists use model documentation and model metadata to determine the maturity of the model and, thus, what level of supporting resources to assign to the model. For example, a mature model’s documentation would include the high-level content described elsewhere in this section, detailed installation and execution notes, and recommendations on input datasets or settings for several example scenarios of importance. Assuming sufficiently rich model metadata—archivists might also provide support with intensive curation, user interface design, and dedicated funding for model executions at the model developer’s institution as requested by the community. Conversely, a less mature model may only be preserved for community reference (e.g., a Zenodo deposit).
- Other model developers benefit from documentation. For example, justifications of decisions that cannot be captured in metadata often help with a model’s replicability. Model developers may also find themselves working across different domains throughout their careers. For this purpose, more accessible documentation may help those who are new to a model’s domain to better understand the model’s nuances. An example is Earth System Documentation, which provides comprehensive documentation about the complex earth system models developed by more than 40 modeling groups worldwide, thereby allowing model developers to better understand the internationally coordinated effort. Additionally, integrating narrative and code through computational notebooks—such as Jupyter—can aid readers and users understand and reuse models. These notebooks interweave explanatory text with executable code, clarifying the model’s functionality and application.

Sharing models with accessible documentation is consistent with, and enabling of, common publication practices. Indeed, accessible documentation helps with a model’s assessment at the peer review stage before publication. Consider that there may be very few peer reviewers for any given submission who have both the relevant domain knowledge and the necessary model development expertise to evaluate a model. Therefore, it is helpful to additionally provide accessible documentation on a model’s parameters and dependencies—perhaps in a README file—that allows for its replicability [42].

### Rule 5: Embrace FAIR principles for sharing models

#### The FAIR principles have gained significant traction throughout open science initiatives. It is important that you don’t try to reinvent the wheel when it comes to good model-sharing practices. With this, you can draw on the FAIR principles when sharing information about your model in its metadata

The principles for creating FAIR metadata for data and software generally apply to how we share computational models. However, models present unique challenges due to their integration of conceptual constructs, metadata and software: models are distinct from the usual target of the FAIR principles. For example, a data set’s metadata does not provide insights about its individual components (i.e., data points), while a model’s metadata may provide insights about some components (e.g., an ML model’s metadata describing distinctions between its training and testing data). Furthermore, when sharing a model’s metadata, we are not necessarily sharing the data on which they were built, calibrated or validated. Rather, we are sharing information *about* the model.

These unique features render the FAIR principles a substantively complex topic for their implementation in the context of models. In what follows, we provide only simple considerations to improve a model’s findability, accessibility, interoperability and reusability, as well as its provenance.

- *Findability*: Models and their related artifacts (e.g., data sets and software) can be found and identified through relevant indexes and repositories if each are assigned persistent identifiers (PIDs) such as digital object identifiers (DOIs) for each version. This also helps situate models within complex knowledge graphs, supporting to findability from a variety of access points (e.g., publication and data set landing pages, internet browsers, and community-specific search interfaces). In many cases, the complex landscape of artifacts relevant to a given model is more properly addressed with a metadata container identifier. For this, we may use a Research Activity Identifier (RAiD) or a Research Object Crate (RO-Crate) [43], which effectively interlink a model’s heterogeneous research elements. Metadata underpinning the chosen PID may also make the model citable in publications or on websites, providing another important component of findability.
- *Accessibility*: When sharing models, a working link to access the complete model should be made available to the public, including code, executable files, and other items needed to use the model. However, items that are restricted by national or institutional policies or contracts should remain restricted until those conditions can be changed. Given the commonality of such situations, model accessibility should be planned ahead of time.
- *Interoperability*: Metadata produced in standard formats can be used to locate models within their broader research contexts, thereby allowing the model’s metadata to be interoperable with other domain-specific research projects. An example of this is at BioModels, EMBL-EBI, where the team of model curators enrich metadata and convert code into standard formats for their respective domains (e.g., systems biology markup language “SMBL” for systems biology, and Open Neural Network Exchange “ONNX” for ML-trained models) [44]. Ultimately, the curators ensure that models made available on the BioModels platform are interoperable with relevant systems and can be adequately indexed by other databases.
- *Reusability*: Model reusability can be enabled through 3 lines of action. Firstly, the usage license for the model must be included in the model’s metadata for the user to understand their legal rights to reuse the model, ideally indicated with a machine-actionable identifier (e.g., from spdx.org). Secondly, metadata can capture information needed to run the model. For example, in some domains, models may only be usable by interacting with code or tools that require complex workflows, such as specialized hardware, programming languages, and other specific dependencies. Consider a model trained to run on a GPU and that requires using the c++/CUDA programming language. In turn, this programming language’s functionality may depend on a certain programming package, such as cuda-toolkits [45]. Finally, models may contain sensitive items that are not publicly available. Such cases emphasize the importance of providing relevant model metadata for their potential reuse when said items become available.
- *Provenance*: Although provenance is a component of reusability (R1.3. [5]), we separate this concept out due to its complexity for models. Provenance refers to the need to (i) explain the history of components for the credibility of a model [46]; (ii) understand the process by which a model produces results in general and for a specific run; and (iii) link all relevant data sets, publications, and other software to the model, such as input and output data or training data [47]. Attribution metadata is useful to understand from where training data was derived (and, if relevant, where they were stored) and/or what base models may also be relevant. For example, image classification models may need to address questions of attribution, licensing (for images), and other provenance-related concerns. Documenting provenance is also a critical component of model validation studies where different versions or frameworks of the model may be used to predict various events with widely varying accuracies.

In models that produce research artifacts such as data, the practices described above must also be included in—or linked from—those artifacts. Although we have focused on making models FAIR, we should also seek the appropriate mechanisms to make model outputs just as FAIR.

Readability and actionability are enabled by the FAIR principles. Model metadata becomes machine readable and actionable when it is aligned with international and community-specific metadata structures. Said alignment enables a wider application of search tools to improve findability, and can improve the richness of the metadata, benefiting all interested stakeholders. The main international metadata structures are DataCite, Schema.org, and CITATION.cff, and should be used to structure the minimal metadata for models. Additional metadata beyond the minimum requirement for PID creation—such as those recommended by CodeMeta—are useful to support discoverability. We should also incorporate community-specific recommendations for FAIR implementation, which often relate to unique disciplines (e.g., the “Data, Optimization, Model and Evaluation” (DOME) [48] recommendations in the bioinformatics community).

### Rule 6: Publicly recognize and reward research software engineers

#### RSEs can play important roles in modeling projects but have only garnered attention in recent years. With RSEs’ varied contributions to modeling, it is important to specifically recognize and reward their work

**A**cknowledging diverse contributions is key to good model-sharing practices. However, there is one key role that is particularly crucial to the modeling process: the RSE. Computational models have always required software of some sort, but the term “RSE” was only coined in 2012, when a group of scholars met in Oxford, to ask: why is there no career for software developers in academia? [49]. Through campaigning and public outreach, the recognition of RSEs gained overwhelming support, and now encompasses about 10,000 professionals worldwide, and 9 region-specific communities [50]. But we must continue to celebrate the work of RSEs, and the value they bring to modeling is varied indeed.

In modeling, RSEs help domain experts work with the complex software they develop. They play a translational role between domain-specific expertise and software-specific tasks, such as data processing, model simulation, and software testing [51]. Thus, the RSE both applies their skills in software engineering, and comes to learn some of the intricacies of the domains with which they engage.

What’s more, RSEs provide a service to domain experts, who often have widely varying degrees of coding, software, and modeling skills. For RSEs, this means having to meet their different users’ specific needs. Some users may need complex cyberinfrastructure solutions to lead projects carried out by entire teams. These users will likely be more tech savvy. At the other end of the spectrum, we may have early career researchers with little technical expertise who require support with simpler tasks.

One approach that has been found to help RSEs tailor solutions to specific user needs is “design thinking,” which is user-centric and solution-based [52]. Software engineers in business settings have applied design thinking for over a decade [53], and it has already been found to relieve tensions in consortia involving both private companies and research institutions [54]. One specific instantiation of design thinking applied by RSEs is at the University of Notre Dame’s Center for Research Computing, where they specifically use this approach to deliver solutions that meet the needs of their users [55].

RSEs also help other stakeholders engage with model outputs in an accessible way. In clinical settings, it has been observed that RSEs remove technical barriers for clinicians and industry partners [56]. The different metadata and documentation resulting from good model-sharing practices also require RSEs’ input, and we have already seen the varied audiences supported by such practices.

With all this, RSEs play a critical role in modeling, and must be given the credit they deserve to attract and retain their talent for modeling purposes. Beyond giving RSEs credit, a good model-sharing practice is to standardize their roles and enable their career development [57]. This requires a more fundamental shift in how RSEs are evaluated in the workplace, as applying traditional academic norms to this emerging role is driving them away from research contexts and depleting the sciences of the skills and experience needed to tackle complex problems [58].

### Rule 7: Deploy user-friendly tools for collaborative modeling practices

#### User-friendly tools, such as interfaces where users can request a model execution, are essential for models to be more seamlessly adopted by domain experts, including those who may lack the specialized computer science training [59]. Models often rely on complex cyberinfrastructure, and science gateways offer a solution for making modeling more accessible and straightforward

In the early 2000s, several initiatives aimed to democratize cyberinfrastructure, making it available to researchers regardless of their geographical location. Through online portals, these initiatives enabled researchers to access various software applications, store and share large data sets, and even obtain training materials. This era marked the beginning of widespread accessibility to computational resources, laying the groundwork for the development of modern science gateways [60].

Today, science gateways provide intuitive interfaces that abstract the underlying technical complexities, enabling researchers to focus on their scientific problems rather than on the intricacies of the software. By offering integrated environments that support various workflows—from data analysis to simulation and visualization—science gateways streamline research processes, foster collaboration, and accelerate discovery. They also facilitate reproducibility and transparency in research by providing standardized tools and methodologies, making it easier for experts to validate and build upon each other’s work. Ultimately, user-friendly science gateways democratize access to advanced computational resources, empowering domain experts to leverage cutting-edge technologies to advance their fields. However, limitations still exist for science gateways to incorporate computationally complex models—such as earth system or forecasting models—requiring the participation of model developers to perform the set-up and execution of the model to create the desired output. Additionally, model coupling requires a human-in-the-loop interaction that needs RSEs to prepare the science gateway for such steps.

One example of a widely used science gateway is MyGeoHub [61]. MyGeoHub allows access to several science gateways for geospatial research and models. One of the platforms that are accessible via MyGeoHub is the WaterHub [62], which provides substantial benefits for Soil and Water Assessment Tool (SWAT) [63] modeling by enhancing open data access and reproducible workflows.

As a centralized repository for SWAT models, MyGeoHub simplifies the process by eliminating the need for complex local setups on a user’s computer for running the models. Furthermore, the science gateway fosters a collaborative environment by allowing users to share data either publicly or within projects they can configure and add members to. MyGeoHub allows for fine-grained security in the different science gateways that people can decide to keep data first private or to share data within a project or fully publicly.

MyGeoHub’s cloud-based scalability and high-performance capability allow users to run complex simulations on powerful resources, thus overcoming hardware limitations. Moreover, the potential integration with visualization tools within MyGeoHub enhances the clarity and communication of research findings. Additionally, MyGeoHub’s compatibility with SWATShare streamlines simulation processes and facilitates easy access to results.

**Fig 2:**
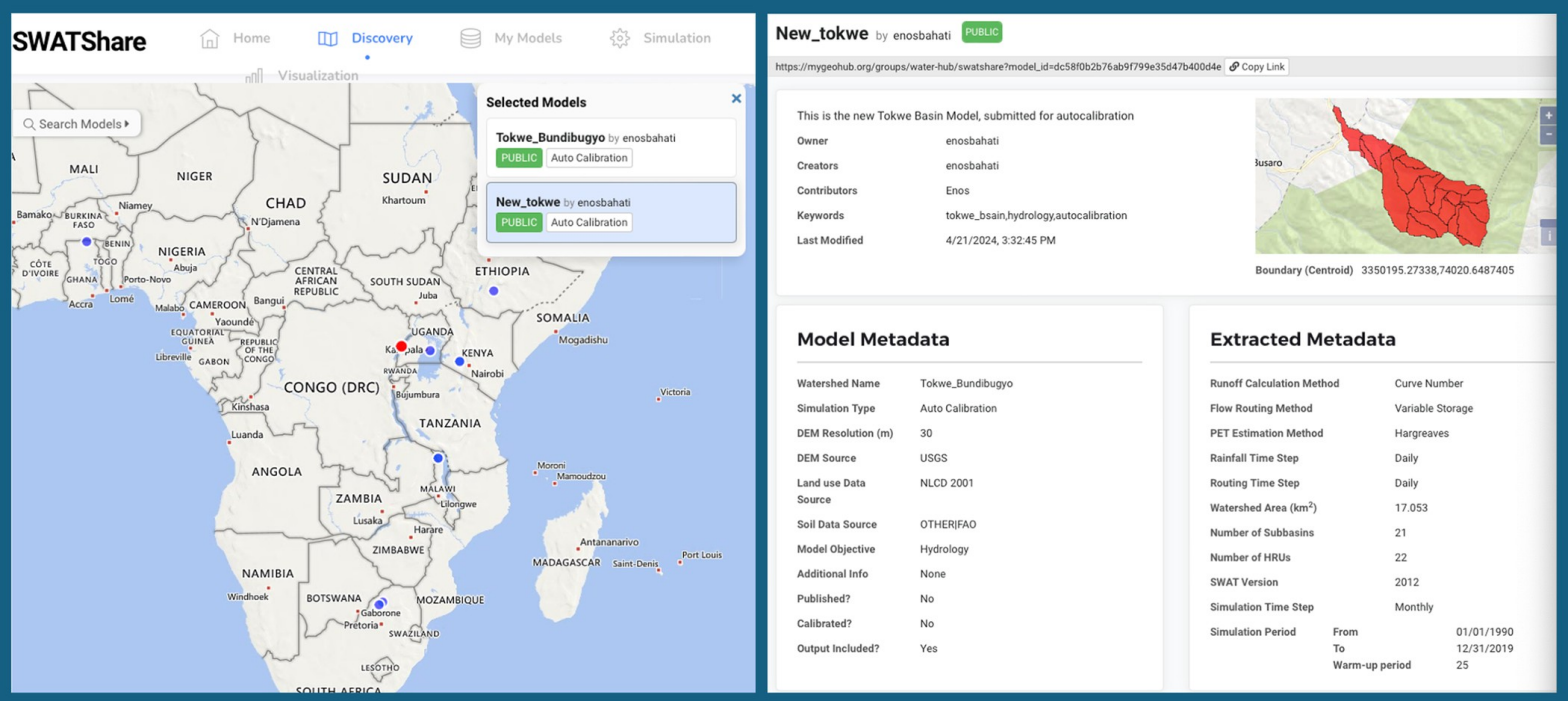
Screenshots from SWATShare. The left panel shows available research projects in blue across most of Africa. The dot in red in Uganda has been selected, and relates with 2 models by Enos Bahati. The right panel shows metadata from the “New_tokwe” model. The figure is available under CC-BY 4.0 and can be replicated at https://mygeohub.org/groups/water-hub/swatshare?model_id=dc58f0b2b76ab9f799e35d47b400d4e.

SWATShare [64] is a web platform of the widely used, public domain hydrologic model SWAT. This semi-distributed, conceptual model simulates various processes, including rainfall-runoff, non-point source pollution, and the impacts of agricultural management practices on watersheds. Developed and maintained by the USDA Agricultural Research Service [65], SWAT is a valuable tool for researchers worldwide. The widespread adoption of SWAT is well-aligned with MyGeoHub’s collaborative framework, as SWAT’s open-source nature complements MyGeoHub’s mission to promote open science practices in hydrology. Fig 2 shows an example of a model created for hydrology research on the Tokwe Watershed by Enos Bahati and shared via SWATShare. This visualization is accessible via the WaterHub in MyGeoHub. In addition to the visualization, users have access to the metadata for this model including important research measures such as simulation time steps and simulation periods—this case, 30 years (Fig 2).

Supporting and training staff, including RSEs, and implementing practices like hallway testing (asking others to use your code to understand any usability issues) [66] are critical practices for ensuring the user-friendliness of services and tools. They help fill information gaps, connect users with complementary tools and services, and facilitate the enhancement and iterative improvement of model interfaces. User-friendly services encompass a range of elements, including effective documentation, comprehensive usage instructions, and demonstration resources. These components collectively enable users to understand and effectively integrate models into their own work, thereby enhancing overall usability and accessibility.

The development of user-friendly tools and the support structures surrounding them are pivotal in making advanced modeling and computational resources accessible to a broader range of researchers. By lowering the barriers to entry, science gateways not only democratize technology but also pave the way for significant scientific advancements, driving innovation and progress across various domains.

Of course, not all research domains have science gateways in place. In such cases, it is worth going back to basics by learning from the earlier initiatives; for example, by pursuing a distributed modeling network approach where modeling capabilities are hosted at different institutions but accessible through a single interface. You can also advocate for science gateways as a possible solution to your community’s needs.

### Rule 8: Influence publishers to promote good model-sharing practices

#### As part of the research community, you have the power to influence what model-sharing practices are valued and adopted. Whichever your role in modeling, you should guide your peers and future scholars towards good model-sharing practices, and seek opportunities to inform the policies that publishers implement

When publishing computational models, it is crucial to adhere to high standards for data- and model-linking by using appropriate PIDs. Detailed metadata, precise version control, and comprehensive documentation of data sources and model parameters are also essential components. These measures enable other scientists to understand and replicate models.

Furthermore, high standards in data- and model-linking facilitate meaningful comparisons across studies and foster collaborative advancements in the field. By maintaining robust links between data and models, researchers can more easily integrate their work with existing studies, leading to new insights and innovations. This approach enhances the credibility and impact of individual research projects, and contributes to the broader scientific community’s collective knowledge.

But who creates standards for model-linking? Generally, there are 2 approaches to developing standards: top-down and bottom-up. Top-down standards may come in the form of governmental or organizational policies. Policy-based approaches have been shown to be the most effective at increasing data-sharing [67], but the degree to which publishers require models to be shared is variable. For example, in a study of 7,500 articles on individual- and agent-based models that were published across 1,500 different journals, only 11% were found to share code [68]. Furthermore, data from DataCite shows that, out of over 17,000 models accessible through their platform, there are less than 1,000 citations in the literature to the models because they are not cited in their related metadata or publications [69].

One instance of a top-down policy can be found at the journal Springer Nature, which now has a policy that encourages code-sharing [70]. The policy’s implementation has relied on the human support and technological infrastructure made available to authors. Indeed, the policy requires journal staff to assist authors in making their code executable on CodeOcean. The policy also results from a pilot, where authors responded with little resistance [71]. This points to the second approach to developing standards.

A bottom-up approach to developing standards for model-linking relies on communities reaching a sort of “tipping point” [72]. At this tipping point, model developers and stakeholders cohesively adopt and disseminate some practice to such a degree that it becomes a standard. We already saw how this might be achieved with the case of the ODD protocol. In the case of Springer Nature’s policy, the research community’s readiness to follow open science practices was instrumental for its viability. With this, there is potential for model developers to promote model-sharing by influencing publication policies [73].

Researchers can shape model-sharing practices in various ways. Those who serve on journal boards can use their positions to influence journal policies. Where this isn’t possible, researchers in their role of peer reviewers can hold others to account and use their position to normalize model-sharing. In addition, community members in educational settings can train budding scientists to share models. The goal is for the next generation of scientists—who will go on to become future editors, reviewers, principal investigators, and teachers—to carry the torch of model-sharing [74].

With this, model-sharing isn’t just about enacting good practices, it’s also about promoting them. Whether you’re a mentor, professor, librarian, PhD candidate, or somebody else in the modeling world, you can inform how your networks approach model-sharing. Being part of academic publication processes is just one way to do this.

### Rule 9: Break down silos

#### Working within silos is detrimental to scientific progress. With modeling being inherently multidisciplinary, you should value the role of collaborating across disciplines and organizations as central to all good model-sharing practices. After all, computational models are shared by people, for scientific and policy advancement, via platforms

The concept of multidisciplinary collaboration in modeling is not new. For example, geoscience requires a diverse collection of expertise including physics, biology, chemistry, and social sciences to model the entire Earth system. This has led to a community-driven approach to model development and sharing called Community Earth System Model, which originated from the United States and the EC-Earth consortium in Europe. This approach is now co-developed by the international community and shared across the globe. Recent technological developments such as cloud computing also make sharing and international collaboration more streamlined.

We have already seen the various stakeholders who benefit from model-sharing practices—from policy makers to publishers and scientists (rule 4). The rule on accessible documentation proffered one approach to break down silos: it is a good model-sharing practice to enable diverse audiences to engage with your models by anticipating their needs through documentation. Indeed, when it comes to modeling for public policy purposes, research has found that involving different stakeholders from the outset increases the likelihood of a model being used and effective [75].

The rule on community-driven insights (rule 2) is also relevant: model developers should establish or be part of communities that encompass diverse disciplines and perspectives. Such communities may even bring together different modeling stakeholders. A key learning rule 2 is that breaking down silos does not happen automatically. Building communities takes effort and must be intentional. Having clear codes of conduct and welcoming different types of contributions are some ways to bring seemingly distinct communities closer together.

The rule on acknowledging diverse contributions (rule 3) further supports the community rule: we are not only engaging with diverse communities but also rewarding them. The CRediT taxonomy we saw there is predicated on the need for different staff—not only academics—to be acknowledged. In this regard, breaking down silos different departments across organizations working cohesively and being recognized in modeling efforts.

We already saw the role RSEs have to play (rule 6), and we can imagine how IT may provide support by making certain hardware and software available; how project managers, events coordinators and communications teams may help a project run smoothly and be effectively disseminated; and how cybersecurity experts may help teams establish policies on what excluded from model-sharing, from passkey values to dependencies on vulnerable packages [76].

Finally, we saw how science gateways are one approach to making cyberinfrastructure accessible to domain experts (rule 7). Increasing the accessibility of models using science gateways further breaks down silos, connecting a greater portion of a given community to advanced modeling capabilities without needing to know the right people.

Collaboration is essential for breaking down silos and fostering innovation in model development. Platforms such as GitHub and Hugging Face are widely used in facilitating the necessary interactions for advancing model-sharing practices [77,78]. The now-forming capability to execute complex models using a mix of cloud and on-premise resources also advances collaboration capabilities, further linking model developer communities together without having to rely on third party platforms to produce a model run [79]. These platforms and capabilities enable dynamic collaboration, allowing for continuous improvement, shared contributions, greater accessibility to both the models and their developers, and new ways to run models.

### Rule 10: Don’t wait for perfection when sharing models

#### As the adage goes: perfection is the enemy of progress. We cannot be paralyzed by the desire to enact every good model-sharing practice each and every time. Rather, we must do the best we can, given the resources we have access to and the policies our institutions implement

Making information about computational models publicly available is typically advantageous. However, there are many elements involved in the process; from clear licensing and different types of documentation to community-building and acknowledging contributions. Although there are challenges that inhibit model-sharing (e.g., a lack of technical skills to create user-friendly interfaces, or time constraints that do not allow for extensive documentation), it is important to realize that the act of sharing a model—in any format—holds more value than withholding it entirely [80–82].

Model-sharing takes place along a spectrum, from minimally making DOIs available to referencing some of a model’s components, to producing comprehensive documentation for diverse stakeholders to learn from a model. With this, it may be difficult to share comprehensive information about models, but we can always consider implementing those simpler, good practices that we *can* achieve.

Actively sharing models can help us be better prepared for the future of modeling. There is, after all, an increasing institutionalization of model-sharing. This is occurring at governmental and organizational levels. The European Union’s AI Act is an example at the government level of increased expectations to share models, whereby models that are components of artificial intelligence (AI) systems may trigger certain exemptions for the model developers if shared for free and open-source [83]. In the US, we find other efforts along these lines, with the White House seeking input on the risks and benefits of making AI models’ weights widely available [84].

At an organizational level, we find various examples of developing and implementing model-sharing policies:

- Wageningen University & Research (WUR) in the Netherlands has a team of model auditors. Their job is to assess the quality of models produced within WUR’s research departments [85,86]. WUR’s Research Modeling Group has also established clear standards for model developers to follow [87,88]. Through their published standards and auditors, model developers at WUR have access to support to ensure their models meet the institutionalized criteria for model-sharing. Moreover, WUR staff have access to their “good modeling practices Wiki,” where they can both learn about standards shared in academic literature and make their own contributions [89].
- The Channing Division of Network Medicine (CDNM) has institutionalized an approach to running code in clinical research settings. In this case, CDNM has developed “Data ID forms” for documenting the location and run date for the code that produces all figures, tables, parameters, and numbers reported in a paper [90]. It is worth noting that this form fits within a wider set of governance structures that promote research integrity.
- NASA, meanwhile, updated their policy regarding the models they fund in 2022. The policy requires models to be shared at the time of publication of the first related article, or at the end of the research award, as long as the software is not restricted. The policy additionally instructs such software to be developed openly on a version-controlled platform. This signals a long-awaited shift towards open science practices for models [91].

As with these 10 simple rules, we cannot expect perfection when sharing models. A variable’s provenance, a discipline’s perspective, or an assumption’s justification may always be missing. We continue to operate in a world where the establishment of standards and availability of peer reviewers for models are far from satisfying. But open modeling practices are becoming institutionalized as the open science movement continues to thrive. With this, you should feel encouraged to publicize your approach to modeling. Only by sharing may you receive community feedback, allow your models to be adopted and adapted by others, and promote good model-sharing practices.
